# Are Endocan Plasma Levels Altered in Preeclampsia?

**DOI:** 10.1055/s-0041-1728661

**Published:** 2021-04-15

**Authors:** Ana Cristina dos Santos Lopes, Suellen Rodrigues Martins, Luci Maria SantAna Dusse, Melina de Barros Pinheiro, Patrícia Nessralla Alpoim

**Affiliations:** 1 Department of Clinical and Toxicological Analysis, Faculdade de Farmácia, Universidade Federal de Minas Gerais, Belo Horizonte, MG, Brazil; 2 Universidade Federal de São Joao del-Rei, Divinópolis, MG, Brazil

Dear Editor,

Our research group has been studying preeclampsia (PE) for over a decade aiming to detect possible blood biomarkers of hemostasis, 1 2 3 4 5 inflammation, 6 7 and endothelial dysfunction 8 9 10 that could be useful for the diagnosis of PE. Until today, only the onset of hypertension (≥ 140 mmHg systolic or ≥ 90 mmHg diastolic) on or after 20 weeks of gestation in association or not with proteinuria and/or evidences of multisystem impairment (such as renal, liver and neurological dysfunctions) is an acceptable criterion to establish the diagnosis of this gestational disease. 11 It is important to emphasize that PE affects between 2 and 8% of all pregnancies worldwide, an early diagnosis of the disease, before the occurrence of systematic impairment, is still not available, which motivates our arduous search for laboratory markers of PE. 12 13

Endocan is a biochemical marker of endothelial dysfunction that is potentially associated with immunoinflammatory response. 14 15 Previous data of our group showed that endothelial dysfunction and inflammation are important features in PE. 6 8 9 Aiming to determine if endocan plasma levels could be useful for determining PE predisposition and/or development, we investigate its levels in preeclamptic and normotensive pregnant women from the southeastern state of Minas Gerais, Brazil.

Our case-control study included 80 Brazilian pregnant women, 40 with severe PE (≥ 160 mmHg systolic or > 110 mmHg diastolic pressure) and 40 normotensive pregnant women (controls). Endocan levels were investigated by enzyme-linked immunosorbent assay (ELISA). The statistical analysis was performed using IBM SPSS Statistics for Windows, version 19.0 (IBM Corp., Armonk, NY, USA). Data normality was tested by the Shapiro-Wilk test. The differences in endocan levels between the PE and normotensive groups were assessed by the Mann-Whitney test. P-values < 0.05 were considered statistically significant.

Surprisingly, no significant difference was observed comparing endocan plasma levels between PE (0.388 ng/mL [0.346–0.516]) and normotensive pregnant women (0.393 ng/mL [0.321–0.623]) ( *p* = 0.870). We classified PE by the onset time of clinical symptoms, such as early (< 34 weeks) or late PE (≥ 34 weeks), 3 and compared endocan levels in these groups. Again, no significant difference was observed for early (0.385 ng/mL [0.311–0.459]) and late PE (0.407 ng/mL [0.313–0.500]) and normotensive pregnant women (0.393 ng/mL [0.244–0.542]) ( *p* = 0.851).

A review of the literature showed eight studies that investigated endocan in women with PE. 16 17 18 19 20 21 22 23 According to our data, two studies showed no significant difference between endocan levels in preeclamptic versus normotensive pregnancy. 16 18 However, five studies revealed increased levels in women with PE versus normotensive pregnancy 17 19 21 22 23 and two studies showed that endocan protein in the placenta tissue is upregulated in PE 18 20 suggesting its involvement in the pathogenesis of PE. Of note, among the studies that found a positive association between endocan levels and PE development, one was also conducted in Brazil. 22 It is well-established that high levels of tumor necrosis factor-α (TNF-α) and vascular endothelial growth factor (VEGF) are able to stimulate the expression of endocan. 24 25 It should be highlighted that the women with PE of the present study showed no previous increase of TNF-α plasma levels 3 and lower VEGF levels 26 comparing with normotensive pregnant. These data could justify why endocan levels were not elevated in the women with PE studied.

Interestingly, Chang et al. 18 related that, although plasma levels of endocan do not correlate with the occurrence of PE, an increased expression of mRNA and endocan were found in the placenta of women with PE, it could suggest that endocan is related to PE pathophysiology, but only in the microenvironment of the placenta, not reflecting the placental changes in plasma. Therefore, our research group aims to evaluate the expression of endocan in the placenta, in addition to repeating the plasma analyzes in a larger sample, to confirm that the results are in fact not significant in the population studied.

In conclusion, our findings showed no association between endocan levels and PE occurrence in Brazilian pregnant women. The role of endocan as endothelial function biomarker is unquestionable. Since endothelial dysfunction and systematic inflammatory response are among the key pathophysiological mechanisms for PE, future studies are required to investigate how endocan is involved in the occurrence of PE.
